# A Fatal Case of Nafcillin-Induced Hepatotoxicity: A Case Report and the Literature Review

**DOI:** 10.1155/2012/953714

**Published:** 2012-07-11

**Authors:** Mian Bilal Alam, Amin Kadoura, Magesh Sathaiah

**Affiliations:** ^1^Department of Internal Medicine, Conemaugh Memorial Medical Center Hospital, Johnstown, PA 15905, USA; ^2^Department of Surgery, University of Pittsburgh School of Medicine, and the University of Pittsburgh Cancer Institute, Pittsburgh, PA 15213, USA

## Abstract

*Background*. Drug-induced hepatotoxicity (DIH) is quite common, and there are several recommendations for its treatment based upon its etiology. DIH may range from mild and subclinical to fulminant liver failure and death. Even though there is extensive list of drugs causing DIH, antibiotics, as a class of drugs, are the most common cause of DIH. Here, we present a fatal case of nafcillin-induced hepatotoxicity confirmed by liver biopsy, with total bilirubin peaking to 21.8 mg/dl and subsequent further extensive evaluation for hepatic injury turning out to be negative.

## 1. Introduction

Nafcillin is a Narrow-spectrum beta-lactam antibiotic used to treat gram-positive staphylococcal infections except for methicillin resistant staphylococcal aureus (MRSA). It is usually a first-line drug for staphylococcal endocarditis in patients without artificial heart valves.It is widely distributed in various body fluids including bile, pleural, amniotic, and synovial fluids with poor CSF penetration but enhanced in meningitis. The mean plasma concentration of the drug was approximately 30 ug/mL at 5 minutes after injection. The serum half-life is 30–60 minutes after intravenous administration of the drug. It is 90% bound to albumin and primarily metabolized in the liver and undergoes enterohepatic circulation.

Nafcillin is a well-tolerated and a commonly prescribed drug for most staphylococcal infections. Adverse drug reactions to this drug range from mild gastrointestinal side effects to severe pseudomembranous colitis with superinfection to *Clostridium difficile*. Agranulocytosis, renal tubular damage, cases of acute interstitial nephritis [1], and acute tubular necrosis [2] have often been reported, but idiosyncratic liver injury with nafcillin is very rare [3]. Hence we report an irreversible fatal case of nafcillin-induced fatal hepatotoxicity with cholestatic jaundice.

## 2. Case Report

**Figure 1:**
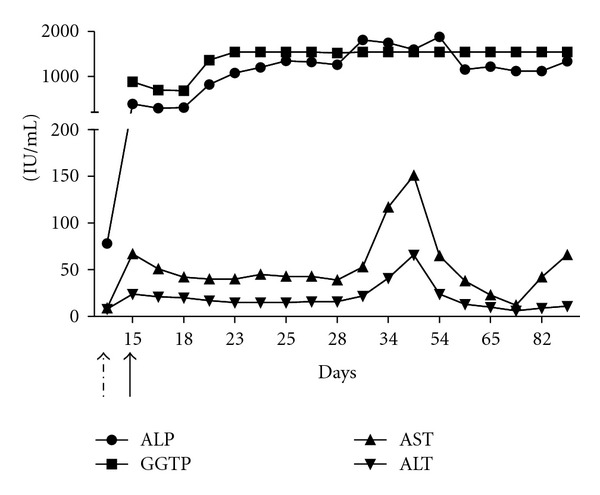
Elevation of AST, ALT, GGTP, and ALP following nafcillin treatment (day 1 marked as dashed arrow). Undashed straight arrow marks the day of nafcillin withdrawal (day 15).

A 53-year-old white female with the past medical history of type II diabetes mellitus, hypertension, hyperlipidemia, depression, coronary artery disease after RCA stent, and peripheral vascular disease after bilateral stents to the common iliac arteries was transferred to our hospital after being diagnosed to have osteomyelitis of right great toe and methicillin-sensitive staphylococcus aureus bacteremia from an outside hospital. She was started on ertapenem for bacteremia before getting transferred to our hospital. The baseline laboratory values done on an outpatient evaluation basis just 10 days prior to the admission were WBC 12.5, hemoglobin 13.4, haematocrit 39, platelets 564, BUN 22, creatinine 1.5, glucose 222, sodium 138 meq/L, potassium 4.2 meq/L, chloride 101 meq/L, bicarbonate 25 meq/L, bilirubin total-0.3 mg/dL, bilirubin direct 0.1 mg/dL, alanine transaminase (ALT)-8 IU/L, aspartate transaminase (AST) 9 IU/L, alkaline phosphatase (ALP) 78 IU/L, direct albumin, 3.8 gm/dL, protein total 7.80 gm/dL, PT-10.2 s, INR 1.0, PTT 28 s, and sedimentation rate 72 h; blood culture showed no growth even after 5 days. On admission, physical examination findings were unremarkable except for her right great toe which was swollen and oozing; pedal pulses were not palpable. Laboratory showed an increase in leukocytes 16,300/uL; reference range (4500–8500), platelets 775 thousand/cu·mm reference (140–440), glucose 300 mg/dL (70–105), creatinine 2 mg/dL (0.6–1.1), low glomerular filtration rate (gfr) 26 mL/min, normal PT 16.4 s (9.0–12.0), PTT 42 s (25–35), and INR 1.6. Blood culture done two times showed no growth even after 5 days. X-ray right foot showed osteomyelitis of right great toe; ertapenem was discontinued and was started on nafcillin 12 gram/day. The day nafcillin started was considered as day 1. Surgery was done under general anesthesia on day 4 for the amputation of right great toe. Abdominal aortogram was performed on day 10 and was found to have mild narrowing at the level of tibioperoneal trunk with areas of stenosis the dorsalis pedis artery was occluded. The patient underwent right second toe amputation and percutaneous angioplasty for worsening of her osteomyelitis. On day 15, she was found to be jaundiced with mild disorientation; her liver function showed elevated total and direct bilirubin, alkaline phosphatase (ALP), and gamma glutamyl transpeptidase (GGTP) levels (bilirubin total (T)-9.6; direct (D)-7.3; ALP-388 IU/L; AST-67 IU/L; ALT-24 IU/L; GGTP-878 IU/L, with decreased creatinine clearance 30 mL/min/24 hr reference range (72–141 mL/min/24 hr). Pharmacy was consulted for drug-related hepatic injury, and nafcillin was discontinued immediately. A mild, transient drop in the levels of AST, ALT, ALP, and GGTP was noted immediately after stopping nafcillin. Further workup for hepatitis and jaundice was done to rule out other causes of hepatotoxicity. Workup included a negative direct coombs, and antibody screening, negative ANCA test for MPO and PR3 antibodies, rheumatoid factor, antismooth muscle antibody, antinuclear antibody, and HLA B27 were also negative. Levels of C3 and C4 complements were normal; liver-kidney microsomal antibody, alpha fetoprotein, and antithyroid peroxidase were also normal. Abdominal ultrasound showed a heterogenous appearance of liver parenchyma, and MRI abdomen was normal. The patient had an elevated GGTP, ALP, and bilirubin, mostly direct bilirubin (Figure 1) before being discharged to transitional care unit for rehabilitation.

On day 36, her jaundice worsened, and she was admitted at a liver transplant center for further evaluation. Her workup included elevated AST 152 IU/L, ALT 85 IU/L, ALP >1800 IU/L, GGT >1500 IU/L, and total bilirubin 11.5 mg/dL, repeat ultrasound abdomen showed no intra- and extrahepatic biliary obstruction; MRCP was unremarkable; liver biopsy showed diffuse hepatocanalicular cholestasis with focal centrizonal bile infarct, periportal hepatocellular swelling and mild nodular hyperplasia like changes. Diagnosis of cholestatic jaundice secondary to nafcillin was made and Ursodiol 10 mg/kg was started. There was no evidence of fulminant hepatic failure, coagulopathy, or encephalopathy. The patient was discharged to home. The patient was followed as an outpatient with regular blood tests for LFT. The GGTP and ALP both remained elevated >1500 IU/L.

On day 54, she was readmitted to the ER for abdominal pain and hematuria. Her workup revealed bilirubin (T-28.7 mg/dL, D-21.3 mg/dL), ALP >1884 IU/L, ALT 24 IU/L, ammonia 45 ug/dL (reference 31–123), creatinine 1.6, INR 5.4, PT 58.5, haemoglobin 7.5 gm/dL (reference 11.5–16.0), and haematocrit 21% (reference 37–47); urinalysis showed blood in urine, and blood culture remained negative. She was transfused two units of blood and was hemodynamically stabilized. She was monitored closely in the hospital. On day 84, she redeveloped an abdominal pain and was shifted to the liver transplant centre for further evaluation, where she went into respiratory arrest and succumbed to her underlying disease conditions.

## 3. Discussion

Nafcillin in general is a safer semisynthetic penicillin antibiotic prescribed most commonly for methicillin-sensitive staphylococcal infections. It is primarily metabolized in liver and excreted unchanged in urine (30%) and predominantly in bile. The most frequently recognized side effect ranges from mild nausea and vomiting to occasional serious *Clostridium difficile* superinfection. Cases of acute interstitial nephritis [1] due to nafcillin and its drug interactions showing the evidence to induce cytochrome P-450 enzymes have been reported with warfarin and nifedipine [4]. The incidence of drug-induced hepatotoxicity [5] and occasionally cases of cholestatic jaundice has been reported with Nafcillin [6]. Although drug-induced cholestasis injury has been reported before, a case of fatal hepatic canalicular injury which never returned to baseline even after discontinuation of the offending drug incites a new warning. The exact mechanism for such toxicities is unclear, but previous reports of hepatic and renal dysfunction with Nafcillin administration greater than 9 g/24 hours [7] suggest an involvement of high-dose therapy as in this case for the treatment of chronic osteomyelitis. Here in this case, the patient had stage III kidney disease secondary to diabetic nephropathy reducing the clearance of Nafcillin and increasing the cholestatic liver injury.

Her laboratory values were consistent with cholestatic jaundice with persistent elevation of ALP and GGTP until her last followup (Figure 1). Extensive workup to rule out other causes of hepatitis and cholestasis, including anti-nuclear antibodies and anti-Sm antibodies, turned out to be negative, ultrasound abdomen was normal, and MRCP was unremarkable. Liver biopsy was consistent with the picture of cholestatic liver injury either drug induced, stricturing, biliary obstruction, or sepsis; since the patient was not septic and blood cultures were negative, and MRCP was unremarkable leaving out with only choice of drug-induced hepatitis. Patient drug list was reviewed with pharmacy and was found to have Nafcillin as the only offending drug to cause cholestasis jaundice.

## 4. Conclusion

Even though nafcillin is a safer drug with fewer side effects, the chances of idiosyncratic drug injury have to be considered. When prescribing higher doses of >10 gram/day, it should be followed up with both renal and liver function tests after initiating such therapies.
